# Supplementary material for: Conflict-related intentional injuries in Baghdad, Iraq, 2003–2014: A modeling study and proposed method for calculating burden of injury in conflict
Source: PLoS Med. 2021 Aug 5;18(8):e1003673. doi: 10.1371/journal.pmed.1003673 (PMC8376016; doi:10.1371/journal.pmed.1003673)
Supplement: S1 STROBE Guidelines Checklist — (PDF) [file pmed.1003673.s001.pdf]

STROBE Statement—Checklist of items that should be included in reports of ***cross-sectional studies***

	Item No	Recommendation	Section and Paragraph #
**Title and abstract**	1	(*a*) Indicate the study's design with a commonly used term in the title or the abstract	Title & 2nd paragraph of Methods Section of Abstract
		(*b*) Provide in the abstract an informative and balanced summary of what was done and what was found	Paragraphs 1,2,3 of Methods and Findings Section of Abstract
**Introduction**			
Background/rationale	2	Explain the scientific background and rationale for the investigation being reported	Paragraphs 3 and 4 of Introduction
Objectives	3	State specific objectives, including any prespecified hypotheses	Paragraph 5 of Introduction
**Methods**			
Study design	4	Present key elements of study design early in the paper	Paragraph 5 of Introduction
Setting	5	Describe the setting, locations, and relevant dates, including periods of recruitment, exposure, follow-up, and data collection	Paragraphs 1,2,3 of Data Collection Subsection of the Methods section
Participants	6	(*a*) Give the eligibility criteria, and the sources and methods of selection of participants	Paragraphs 1 and 6 of the Data Collection Subsection of the Methods Section, Paragraph 1 under Sampling Subsection
Variables	7	Clearly define all outcomes, exposures, predictors, potential confounders, and effect modifiers. Give diagnostic criteria, if applicable	Paragraph 2 of the Data Collection Subsection of the Methods Section
Data sources/ measurement	8*	For each variable of interest, give sources of data and details of methods of assessment (measurement). Describe comparability of assessment methods if there is more than one group	Paragraph 2 of the Data Collection Subsection of the Methods Section and Questionnaire provided as supplementary information
Bias	9	Describe any efforts to address potential sources of bias	Paragraph 1 of the Burden of Injury Methodology, specifically the final sentence, Final paragraph of Burden of Injury Methodology Subsection
Study size	10	Explain how the study size was arrived at	Final sentence of Sampling Subsection
Quantitative variables	11	Explain how quantitative variables were handled in the analyses. If	The subsections on Calculation of YLLs, short term injury, long term injury with recovery (both treated and untreated, long term injury

		applicable, describe which groupings were chosen and why	without recovery (both treated and untreated describe the calculation of all quantitative variables.
Statistical methods	12	(*a*) Describe all statistical methods, including those used to control for confounding	An analysis plan is provided as supplementary data including sample calculations on individual subjects (S1 Analysis Plan). The specific statistical methods are described under the subheadings: Calculation of YLLs, Short term injury, Long term injury with recovery both treated and untreated, Long term injury without recovery both treated and untreated, Use of incidence and prevalence DALYs, Bootstrapping, and Extrapolation to the City of Baghdad
		(*b*) Describe any methods used to examine subgroups and interactions	The last sentence on the first paragraph under Bootstrapping subsection details the sampling methods for cause specific estimates
		(*c*) Explain how missing data were addressed	Paragraph 2 of the Bootstrapping subsection
		(*d*) If applicable, describe analytical methods taking account of sampling strategy	Paragraph 5 of the Results Section details the interaction of analytical methods and sampling strategy
		(*e*) Describe any sensitivity analyses	Paragraph 3 of the Burden of Injury Methodology subsection in the Methods Section
**Results**			
Participants	13*	(a) Report numbers of individuals at each stage of study—eg numbers potentially eligible, examined for eligibility, confirmed eligible, included in the study, completing follow-up, and analysed	Paragraphs 1 and 2 of the Results section
		(b) Give reasons for non-participation at each stage	Paragraph 2 of the Results Section
		(c) Consider use of a flow diagram	We have considered this, and do not feel a flow diagram would substantively add to this work.
Descriptive data	14*	(a) Give characteristics of study participants (eg demographic, clinical, social) and information on exposures and potential confounders	Paragraph 1 Results Section
		(b) Indicate number of participants with missing data for each variable of interest	Paragraph 2 of the results section

Outcome data	15*	Report numbers of outcome events or summary measures	Paragraph 3 of the Results Section
Main results	16	(*a*) Give unadjusted estimates and, if applicable, confounder-adjusted estimates and their precision (eg, 95% confidence interval). Make clear which confounders were adjusted for and why they were included	Paragraphs 3,4,5 of the Results Section
		(*b*) Report category boundaries when continuous variables were categorized	N/a
		(*c*) If relevant, consider translating estimates of relative risk into absolute risk for a meaningful time period	N/a
Other analyses	17	Report other analyses done—eg analyses of subgroups and interactions, and sensitivity analyses	Paragraphs 3,4,5 and Table 1 of the Results Section
**Discussion**			
Key results	18	Summarise key results with reference to study objectives	Paragraph 1 of the Discussion Section
Limitations	19	Discuss limitations of the study, taking into account sources of potential bias or imprecision. Discuss both direction and magnitude of any potential bias	Paragraph 2, 7,8,9,10,11 of the Discussion Section focus on limitations of the methodology presented
Interpretation	20	Give a cautious overall interpretation of results considering objectives, limitations, multiplicity of analyses, results from similar studies, and other relevant evidence	Paragraph 1 & 3 of the Discussion section
Generalisability	21	Discuss the generalisability (external validity) of the study results	Paragraph 5 of the Discussion Section
**Other information**			
Funding	22	Give the source of funding and the role of the funders for the present study and, if applicable, for the original study on which the present article is based	Both the funding sources for this work and for the work on which our modelling are based are declared in the funding header underneath the Title and authors.

*Give information separately for exposed and unexposed groups.

Note: An Explanation and Elaboration article discusses each checklist item and gives methodological background and published examples of transparent reporting. The STROBE checklist is best used in conjunction with this article (freely available on the Web sites of PLoS Medicine at http://www.plosmedicine.org/, Annals of Internal Medicine at http://www.annals.org/, and Epidemiology at http://www.epidem.com/). Information on the STROBE Initiative is available at www.strobe-statement.org.
