# Supplementary material for: Conflict-related intentional injuries in Baghdad, Iraq, 2003–2014: A modeling study and proposed method for calculating burden of injury in conflict
Source: PLoS Med. 2021 Aug 5;18(8):e1003673. doi: 10.1371/journal.pmed.1003673 (PMC8376016; doi:10.1371/journal.pmed.1003673)
Supplement: S1 Analysis Plan — (PDF) [file pmed.1003673.s002.pdf]

Analysis Plan for Conflict-related intentional injuries in Baghdad, Iraq 2003-2014: A modeling study and proposed method for calculating burden of injury in conflict

Specific Aims

Utilize the information made available by Lafta et al. and the GBD methodology, to calculate the total Disability adjusted life years lost to intentional injury from 2003-2014 in Baghdad, Iraq.

Introduction

Several major works have sought to describe the effects of the Iraq war on the local population. Roberts et al. conducted a cluster sample survey of Iraq in order to compare mortality in the 14.6 months leading up to the invasion of Iraq and the 17.8 months following the invasion. They identified a 2.5-fold increase in the risk of death following the invasion, or 1.5-fold increase if a cluster in Fallujah that accounted for two thirds of all deaths was excluded [1]. Due the short time frame of the Roberts study, this work was updated by Burnham et al. in 2006. This follow up study documented a decreased proportion of deaths due to coalition forces compared to the initial study and a rise in the numbers of deaths related to car bombs, although gunshot wounds (GSWs) remained the primary cause of conflict related deaths [2]. This follow up study demonstrated the importance of ongoing surveillance of the effects of conflict. The authors were able to document the true scale of the high-profile changes in the nature of conflict, such as the increase in car bombings that attract media attention, but also demonstrate that some underlying facts had not changed, such as GSWs continuing to be the largest driver of conflict related injury.

The Iraq Family Health Survey provided a separate effort to document the increase in mortality in Iraq during the invasion. This work captured information from 2002 – 2006 in a single study and utilized a survey of over 9000 households to calculate that an excess of 151,000 deaths had occurred due to the invasion, including adjustments for underreporting among survey participants.

The United Nations High Commission for Refugees (UNHCR) estimates that 2.2 million people had fled Iraq by 2007 [3]. Hagopian et al. added to the existing body of work surrounding morbidity and mortality in Iraq by utilizing a two-stage cluster sampling method of 2,000 households throughout the country [4]. Fluctuating population levels during conflict can present a persistent issue in establishing a denominator for population based calculations, particularly when studies are conducted at the country wide level. Dr. Hagopian and her team reviewed demographic data and secondary sources to estimate the number of households who had fled the country and added this number to the total death count captured by the household survey.

Lafta et al. have provided a more recent update to the growing body of work related to the conflict in Iraq [5]. This study is discussed extensively below, but was more narrowly focused than the other works mentioned here, did not adjust for migration, but covered a time period from 2003-2014.

These works have focused primarily on death counts, with some capture of non-fatal injuries and resulting disability, particularly by Dr. Lafta's team. Non-fatal injuries, however, represent an important metric for the effects of conflict on a population and can have long lasting effects. Amputations due to

landmines are the most commonly noted manifestation of this, with individuals never returning to a state of full health prior to being injured as a result of conflict. Indeed, in 2015 conflict related morbidity accounted nearly half as much morbidity burden as road traffic instances, (38.26 DALYs per 100,000 vs. 87.43 DALYs per 100,00) which have long recognized as a major driver of worldwide burden of injury [6].

We propose that the need for more detailed descriptions of the burden of non-fatal injuries and the societal burden of the effects of conflicts on different age groups of the population are the next steps in building on the impressive work of the teams noted above. In order to accomplish this, we have combined the work of Lafta et al. with the methodological approaches for measuring burden of injury and disease of the Global Burden of Disease (GBD) project to provide a description of the burden of intentional injury in Baghdad from 2003 -2014.

Methods

Samples for Pull Methodology

In order to allow for an assessment of the accuracy of future data set wide calculations the initial step for burden of disease calculations was to perform four "Pull" Calculations. Statistical analysis was performed using the R programming language [7]. Four individual subjects were selected from the dataset, which included 5148 patients in total and 225 patients who were recorded as having a mechanism of injury consistent with intentional injury. One individual with mortality due to intentional injury, one individual with an intentional injury and subsequent short term disability, one individual with an intentional injury resulting in long term disability from which the individual subsequently recovered, and one individual with long term disability from which he or she did not subsequently recover. This analysis will be limited to those individuals with conflict related intentional injury. These individuals were selected by identifying all individuals in the data with an intentional injury as well as specific "recovery patterns" then selecting one individual at random from each of the injury patterns. For example, to select an individual with a long-term injury with recovery, all individuals with intentional injury, who did not die from the injury, and with duration of disability longer than 12 months, but without continuing disability will be selected. Then one individual is selected at random from this group to be used for the pull calculation. The survey included specific questions regarding the length of time that injuries persisted as well as the degree of functional impairment from the injury. Past work for calculations by the Global Burden of Disease project (GBD) has included the work required to correlate the *cause of injury*, which is commonly documented in records of traumatic injury, with the *nature of injury* [8, 9]. The cluster randomized survey utilized in this analysis contained questions specific to both the cause and the nature of the injury.

YLLs

Following the methodology from GBD, YLL was calculated using the GBD maximum life table [10]. Following selection of an individual who was noted as having died due to his or her injury, the age at death was compared against the maximum life expectancy table to obtain the YLL due to injury. In the case of the pull calculation for death at the time of injury, study subject 778 died of a gunshot wound (GSW) at age 24. Comparison to the life table shows that a 24-year-old individual has a remaining life expectancy of 62.96 years at the time of death. Thus, this individual contributed 62.96 YLLs to the overall burden of injury for Baghdad between 2003 and 2013.

The life expectancy of an individual in Iraq is shorter than the maximum life expectancy utilized for previous GBD calculations and cited above. The most recent World Bank calculation of life expectancy for Iraq puts the life expectancy at 69.4 years [11]. Repeating this calculation utilizing country specific life expectancy results in 45.4 YLL by this individual.

YLDs

The second portion of the disability adjusted life years (DALY) calculation involves the calculation of years lived with disability (YLDs). YLDs are a crucial part of the complete burden of disease calculation as they allow for the proportion of disease burden due to disability [8, 9, 12-14]. The first step in selecting subjects for pull calculations in the 3 YLD categories was to determine within the Iraq survey data set what constituted a short term and long term injury and if those injuries were treated or not. GBD has published the duration of short term injury suspected for all injury diagnosis codes used in the study [15]. For the purposes of the Iraq data set an injury was said to be short term if the duration of disability captured on the survey was less than the duration of short term injury for a given code as calculated for the GBD burden of injury work and annotated in Section 9 of the Annex to Haagsma et al [15].

Short term injury

In order to select an individual with short term injury, all individuals with disability less than 1 month following an intentional injury were selected and one subject was selected at random by utilizing the random sample function from the R "dplyr" package [16]. This individual was 35 years old at the time of injury and sustained multiple GSW related injuries to the lower leg resulting in a disabled time of 1 week or approximately 0.02 years. The multiple injuries cited correlated most closely to a mapping code of N48 (multiple fractures, dislocations, wounds, and strains). Due to the fact that no single number can adequately calculate the way a disability weight (DW) will affect the life of a given person, disability weights for the GBD 2013 were designed to account for uncertainty [9, 17]. The exact method for generating these is described elsewhere in detail [17]. Simply, there are 1000 slightly differing DW draws for each N code. One draw of the 1000 was selected at random and applied to the 0.02 years of disability experienced by this individual. Therefore, an individual with a disability lasting 0.02 years with a DW of 0.347 (draw 621) results in 0.0067 YLDs contributed by this short-term injury.

Long Term Injury with Recovery

An individual was selected randomly from those subjects with an intentional type injury and who were coded as being currently alive and functioning normally but having had a disability for longer than one month. Additionally, this person was checked to ensure that the duration of injury was longer than the short term injury duration cited in the GBD short term injury tables [15]. This selection process identified an individual who sustained a foot fracture secondary to a GSW with a subsequent 6 months of disability and treatment at a hospital resulting in a procedure without hospitalization. A DW code of N17 was selected (fracture of foot bones except ankle) and a weight was randomly selected from the 1000 draws. The individual with a long term treated injury with subsequent recovery therefore was disabled for 0.5 years with a DW of 0.037 (draw 601), resulting in a contribution of 0.0187 YLDs to the total.

For the purpose of pull calculations this calculation was repeated with a different definition of long term injury in order to hew more closely to the GBD calculations that sets the long term/short term cutoff at one year of disability who subsequently recovered from the injury. This selection process yielded an

individual (study ID = 563) who had an intentional injury resulting in a lower leg fracture. This individual had 2 years of disability but subsequently made a full recovery. The same DW code as above was applied and a random sampling with replacement yielded a DW of 0.024 (draw 892). This resulted in a contribution of 0.048 YLDs.

Long Term Injury Without Recovery

An individual with an intentional type injury and coded as having continuing disability was selected. This process identified an individual who sustained multiple lower leg injuries following a gunshot wound to the lower leg at the age of 29 years with subsequent treatment at a hospital with admission. This was coded as having a DW corresponding to multiple fractures, dislocations, wounds, and strains. One DW was randomly selected from the 1000 draws corresponding to that specific N code (N48). Based on the GBD life table this individual at the age of 29 had a life expectancy of 87.01 years which implies a duration of disability of 87.01 – 29 = 58.01 years [10]. This duration was then multiplied by the selected DW of 0.046 resulting in a total of 2.7 YLDs.

As stated above the life expectancy for an Iraqi citizen is 69.4 years. If this calculation is repeated utilizing this most recent life expectancy at birth from the World Bank, then this individual contributes 1.85 YLDs to the total.

DALYs

For a given population the disability adjusted life years would be totaled by summing the four above mentioned values. Therefore 2.7+0.0187+0.0067+62.96= 65.69 DALYs lost due to the Iraq war in Baghdad by these 4 individuals between 2003 and 2014.

Tables

Table 1. Pull Calculations. The four injury pattern patients chosen at random for hand calculation. Pull DALYs represents the hand calculated column, and Calculated DALYs represents calculation for these specific cases following full data set calculation.

	Study ID	Age at Injury	Injured Part	Cause of Injury	Disability Length	Care	DW Code	DW Language	DW Draw	DALYs
Long Term Disability No Recovery	442	29	Lower Leg	GSW	Disability Continuing	Yes	N48	Multiple Fractures	0.04605	2.7
Short-term Disability	169	25	Lower Leg	GSW	For weeks	Yes	N48	Multiple Fractures	0.1662	0.0067
Long-term Disability with Recovery	563	44	Lower Leg	Other	For years	Yes	N17	Fractures of Foot Bones except Ankle	0.02455	0.048
Death	778	24	Head	GSW	NA	N/A	N/A	N/A	1	62.96

References

1. Roberts L, Lafta R, Garfield R, Khudhairi J, Burnham G. Mortality before and after the 2003 invasion of Iraq: cluster sample survey. Lancet. 2004;364(9448):1857-64.
2. Burnham G, Lafta R, Doocy S, Roberts L. Mortality after the 2003 invasion of Iraq: a cross-sectional cluster sample survey. Lancet. 2006;368(9545):1421-8.
3. UUNR. UN agencies launch $85-million appeal to aid 2.2 million Iraqi refugees online: UN News Centre. http://www.un.org/apps/news/story.asp?NewsID=23851#.VnwbQvkrIdU. 2007
4. Hagopian A, Flaxman AD, Takaro TK, Esa Al Shatari SA, Rajaratnam J, Becker S, et al. Mortality in Iraq associated with the 2003-2011 war and occupation: findings from a national cluster sample survey by the university collaborative Iraq Mortality Study. PLoS Med. 2013;10(10):e1001533. PubMed PMID: 24143140.
5. Lafta R, Al-Shatari S, Cherewick M, Galway L, Mock C, Hagopian A, et al. Data from: Injuries, death, and disability associated with 11 years of conflict in Baghdad, Iraq: a randomized household cluster survey. Dryad Data Repository; 2015.
6. Institute for Health Metrics and Evaluation. GBD Compare, Viz Hub. https://vizhub.healthdata.org/gbd-compare/.
7. R Core Team. R: A language and environment for statistical computing. Vienna, Austria: R Foundation for Statistical Computing; 2013.
8. Vos T, Flaxman AD, Naghavi M, Lozano R, Michaud C, Ezzati M, Shibuya K, Salomon JA, Abdalla S, Aboyans V. Years lived with disability (YLDs) for 1160 sequelae of 289 diseases and injuries 1990–2010: a systematic analysis for the Global Burden of Disease Study 2010. Lancet. 2013;380(9859):2163-96.
9. Salomon JA, Vos T, Hogan DR, Gagnon M, Naghavi M, Mokdad A, Begum N, Shah R, Karyana M, Kosen S. Common values in assessing health outcomes from disease and injury: disability weights measurement study for the Global Burden of Disease Study 2010. Lancet. 2013;380(9859):2129-43.
10. Naghavi M, Wang H, Lozano R, Davis A, Liang X, Zhou M, Vollset SE, Ozgoren AA, Abdalla S, Abd-Allah F. Global, regional, and national age-sex specific all-cause and cause-specific mortality for 240 causes of death, 1990-2013: a systematic analysis for the Global Burden of Disease Study 2013. Lancet. 2015;385(9963):117-71.
11. World Bank Country Data: Iraq Online: World Bank; 2016 [cited 7 Jan 2017].
12. Murray CJ, Lopez AD. Global health statistics: a compendium of incidence, prevalence and mortality estimates for over 200 conditions. Harvard University Press; 1996.
13. Murray CJ. Rethinking DALYs. The global burden of disease. 1996;1:1-98.
14. Murray CJ, Ezzati M, Flaxman AD, Lim S, Lozano R, Michaud C, et al. GBD 2010: design, definitions, and metrics. Lancet. 2012;380(9859):2063-6.
15. Haagsma JA, Graetz N, Bolliger I, Naghavi M, Higashi H, Mullany EC, Abera SF, Abraham JP, Adofo K, Alsharif U. The global burden of injury: incidence, mortality, disability-adjusted life years and time trends from the Global Burden of Disease study 2013. Inj Prev. 2016 Feb 1;22(1):3-18.
16. Wickham H, Francois R. dplyr: A grammar of data manipulation. R package version 04. 2015;1:20.
17. Salomon JA, Haagsma JA, Davis A, de Noordhout CM, Polinder S, Havelaar AH, et al. Disability weights for the Global Burden of Disease 2013 study. Lancet Glob Health. 2015;3(11):e712-e23.
