# Supplementary material for: Conflict-related intentional injuries in Baghdad, Iraq, 2003–2014: A modeling study and proposed method for calculating burden of injury in conflict
Source: PLoS Med. 2021 Aug 5;18(8):e1003673. doi: 10.1371/journal.pmed.1003673 (PMC8376016; doi:10.1371/journal.pmed.1003673)
Supplement: S1 Data Dictionary and R code Publication — (PDF) [file pmed.1003673.s003.pdf]

Calculating the burden of intentional injuries and disabilities in Baghdad, 2003-2014

Jensen G.W., Lafta R., Burnham G., Hagopian A., Simon N., Flaxman A.D.

Data Dictionary and R Code Publication

1. *study ID* – Numeric variable for study ID of subjects
2. *intentionaltype* – Character variable for mechanism of intentional injury
3. *injuredage* – Numeric variable for age of subject at time of injury
4. *wasinjdeath* – Factor variable with 3 levels, “Death due to injury”, “Death maybe due to injury”, “Death unrelated to injury” Denoting whether an injury resulted in death
5. *part1* – Character variable for primary injured body part
6. *part2* – Character variable for secondary injured body part
7. *disabledlength* – Numerical variable for length of disability in fractions of years
8. *howlongdiscat* – Factor variable for general length of disability, “For days”, “For weeks”, “For months”, “For years”, “Disability is continuing”
9. *wherecare* – Factor variable for location of immediate post injury care, used in analysis as a binary variable for whether injury was classified as treated or untreated. Factor levels include: “Hospital”, “PHC”, “Work or Private Clinic”, “Nurse/other”, “No care”
10. *IYLs* – Character value for number of years remaining until predicted live expectancy based on Iraqi life expectancy
11. *Pred_ex* – Character value for number of years remaining until predicted life expectancy based on GBD life expectancy table
12. *MN-mapping for N* – Disability weight code for each injury
13. *I_DALYs* – DALYs lost per subject based on Iraqi life expectancy, estimated with a single disability weight (DW) draw
14. *Asp_DALYs* – DALYs lost per subject based on aspirational life expectancy, estimated with a single DW draw

Packages utilized for R(1) analysis

dplyr(2)

R Code for bootstrapped analysis for estimation of DALYs lost due to intentional injury in Baghdad

Dictionary of terms within function

1. no_intentional – subsetting of all patients in each bootstrapped repetition of the dataset without intentional injury
2. short_disability - subsetting of all patients in each bootstrapped repetition of the dataset short term disability from injury
3. die_disability – subsetting of patients who died due to injury
4. long_disability_no_care_continuing - subsetting of all patients in each bootstrapped repetition of the data set with long term disability without recovery who did not receive care at the point of injury
5. long_disability_no_care_year - subsetting of all patients in each bootstrapped repetition of the data set with long term disability who recovered and did not receive care at the time of injury
6. long_disability_care_year - subsetting of all patients in each bootstrapped repetition of the data set with long term disability who recovered and received care at the time of injury
7. long_disability_care_continuing - subsetting of all patients in each bootstrapped repetition of the data set with long term disability who did not recover and received care at the time of injury
8. draw_T_DW – DWs for patients with long term injury who received care at the time of injury

9. draw_UT_DW – DWs for patients with long term injury who did not receive care at the time of injury
10. draw_ST_DW – DWs for patients with short term injury, both treated and untreated
11. LDCC_rand – matching of long term disability without recovery who received care with DW from the drawselect function
12. LDCY_rand - matching of long term disability with recovery who received care with DW from the appropriate drawselect function
13. LDNC_rand - matching of long term disability without recovery who did not receive care with DW from the appropriate drawselect function
14. SD_rand - matching of short term disability with DW from the appropriate drawselect function
    - It should be noted that no patients in the dataset fit the pattern of long term injury who recovered and did not receive care, thus there was no need for a matching function for those individuals.
15.

```
DALY_cal_total_all_p = function(x){

  repeat{

    n = dim(x)[1]

    m = sample(1:n, size=n, replace=TRUE)

    b.sample = x[m,]


#Subsetting of injury patterns from the dataset

   no_intentional<- b.sample %>% filter(is.na(intentionaltype))


 short_disability <- b.sample %>% filter(!is.na(intentionaltype)) %>%

      filter((howlongdiscat %in% c("For days", "For weeks", "For months")) & !is.na(howlongdiscat) &
!is.na(wherecare) & (wasinjdeath!="Death due to Injury" | is.na(wasinjdeath)))


     die_disability <- b.sample %>% filter(!is.na(intentionaltype)) %>%

      filter(wasinjdeath == "Death due to Injury")


     long_disability_no_care_continuing <- b.sample %>% filter(!is.na(intentionaltype)) %>%
filter(howlongdiscat=="Disability is continuing" & !is.na(howlongdiscat) & !is.na(wherecare) & wherecare=="No
Care" & (wasinjdeath!="Death due to Injury" | is.na(wasinjdeath)))


 long_disability_no_care_year <- b.sample %>% filter(!is.na(intentionaltype)) %>%

      filter(howlongdiscat=="For years" & !is.na(howlongdiscat) & !is.na(wherecare) & wherecare=="No Care" &
(wasinjdeath!="Death due to Injury" | is.na(wasinjdeath)))


      long_disability_care_year <- b.sample %>% filter(!is.na(intentionaltype)) %>%
```

```
      filter(howlongdiscat=="For years" & !is.na(howlongdiscat) & !is.na(wherecare) & !(wherecare=="No Care") & (wasinjdeath!="Death due to Injury" | is.na(wasinjdeath)))

    long_disability_care_continuing <- b.sample %>% filter(!is.na(intentionaltype)) %>%
      filter(howlongdiscat=="Disability is continuing" & !is.na(howlongdiscat) & !is.na(wherecare) & !(wherecare=="No Care") & (wasinjdeath!="Death due to Injury" | is.na(wasinjdeath)))



    if(nrow(short_disability)!=0 & nrow(die_disability)!=0 & nrow(long_disability_no_care_continuing)!=0 & nrow(long_disability_care_year)!=0 & nrow(long_disability_care_continuing)!=0)break
  }


  repeat{
    # draw the random columns of each disability weight value, so that DWs vary across each repetition of the equation
    draw_T_DW<-drawselect(LTTW.2.Append) # DWs for long term disability both in years and continuing who received care
    draw_UT_DW<-drawselect(LTNTW.2.Appended) #DWs for long term disability who did not recieve care
    draw_ST_DW<-drawselect(shorttermweights) # DWs for short term disabilities


    # This step selects the appropriate disability weight codes on each variation of the equation to be matched with the actual data that has been sampled with replacement on each repetition


    LDCC_rand = draw_T_DW[match(long_disability_care_continuing$`MN-mapping_for_N`, draw_T_DW$`MN-mapping_for_N`), 2]


    LDCY_rand = draw_T_DW[match(long_disability_care_year$`MN-mapping_for_N`, draw_T_DW$`MN-mapping_for_N`), 2]


    LDNC_rand = draw_UT_DW[match(long_disability_no_care_continuing$`MN-mapping_for_N`, draw_UT_DW$`MN-mapping_for_N`), 2]


    SD_rand = draw_ST_DW[match(short_disability$`MN-mapping_for_N`, draw_ST_DW$`MN-mapping_for_N`), 2]



    if(length(SD_rand)!=0 & length(LDNC_rand)!=0 & length(LDCY_rand)!=0 & length(LDCC_rand)!=0) break
```

```
  }
```

#This step allows the DWs for the individual repetition of the function to be matched to the sampled study subjects after being matched to the disability weight code in the previous step

```
  long_disability_care_continuing = cbind(long_disability_care_continuing, LDCC_rand)

  long_disability_care_year = cbind(long_disability_care_year, LDCY_rand)

  long_disability_no_care_continuing = cbind(long_disability_no_care_continuing, LDNC_rand)

  short_disability = cbind(short_disability, SD_rand)
```

#Our analysis looked only at intentional injuries therefore, the DALY contribution for unintentional injuries that fit the given injury patterns as 0. The DW weight and/or life expectancy at this point for these patients without intentional injuries is NA. This steps sets the NAs to 0s, so that the DALY contribution is 0 for those patients without intentional injury.

```
  long_disability_care_continuing<- long_disability_care_continuing %>% replace_na(list(LDCC_rand = 0))

  long_disability_care_year<-long_disability_care_year %>%  replace_na(list(LDCY_rand=0))

  long_disability_no_care_continuing<-long_disability_no_care_continuing %>% replace_na(list(LDNC_rand=0))

  short_disability<-short_disability %>%  replace_na(list(SD_rand=0))

  die_disability<-die_disability %>% replace_na(list(Pred_ex=0))

  no_intentional<- no_intentional %>% replace_na(list(Pred_ex=0))
```

#Calculation of DALYs for each injury pattern on each repetition, from first to last: Individuals with long term disability without recovery who received care = remaining life expectancy multiplied by DW, individuals with long term disability who received care and recovered = length of disability in fractions of years multiplied by DW, individuals with long term disability who did not receive care and did not recover = remaining life expectancy multiplied by appropriate DW, individuals with short term disability = disabled length in fractions of a year multiplied by an appropriate DW, individuals who died of injury = remaining life expectancy, individuals who died of unintentional injuries = remaining life expectancy (set to 0 in previous step)

```
  DALY.allp = c(with(long_disability_care_continuing, Pred_ex*LDCC_rand),

               with(long_disability_care_year, disabledlength*LDCY_rand),

               with(long_disability_no_care_continuing, Pred_ex*LDNC_rand),

               with(short_disability, disabledlength*SD_rand),

               die_disability$Pred_ex,no_intentional$Pred_ex)
```

#converting the data into summary statistics and a data frame

```
  out.allp = data.frame(mean = mean(DALY.allp, na.rm=TRUE),
```

```
                sd = sd(DALY.allp, na.rm=TRUE), sum = sum(DALY.allp, na.rm=TRUE),
                min = min(DALY.allp, na.rm=TRUE), Q1 = quantile(DALY.allp, probs=0.25,
                                    na.rm=TRUE), median = median(DALY.allp, na.rm=TRUE),
                Q3 = quantile(DALY.allp, probs=0.75, na.rm=TRUE),
                max = max(DALY.allp, na.rm=TRUE))



  return(out.allp)



}
``
```

#use of sapply function within R to repeat the function 1000 times for bootstrapping

```
set.seed(1)
out.allp = data.frame(t(sapply(1:1000, function(x) DALY_cal_total_all_p(DALY_whole))))
out2.allp = data.frame(sapply(1:dim(out.allp)[2], function(x) unlist(out.allp[,x])))
names(out2.allp) = names(out.allp)
```

#Reporting of summary statistics

```
apply(out2.allp, 2, mean)
apply(out2.allp, 2, sd)
apply(out2.allp,2, summary)

quantile(out2.allp$sum, probs = 0.025)
quantile(out2.allp$sum, probs = 0.975)
```

*Note drawselect is a custom function for randomly selecting DW draws

# Displayed here is the drawselect function that is embedded in the function above. Each of the 3 sets of DWs contains a row for each DW code and 1000 columns with a normal distribution of DWs for each code. This function selects a column at random and allows for matching of the value based on the DW code.

Drawselect function

```
drawselect<-function(x) {
  samplepick<-sample(2:1001,1)
```

```
  select(x,1,num_range("draw",samplepick))
}
```

References

1. Team RC. R: A language and environment for statistical computing. R Foundation for Statistical Computing; 2015.
2. Wickham H, Francois R. dplyr: A grammar of data manipulation. R package version 04. 2015;1:20.
