# Supplementary material for: Conflict-related intentional injuries in Baghdad, Iraq, 2003–2014: A modeling study and proposed method for calculating burden of injury in conflict
Source: PLoS Med. 2021 Aug 5;18(8):e1003673. doi: 10.1371/journal.pmed.1003673 (PMC8376016; doi:10.1371/journal.pmed.1003673)
Supplement: S1 Questionnaire — (PDF) [file pmed.1003673.s004.pdf]

Iraq Household injury survey 2014

Date_________________ Cluster no ____________ Interviewer code________

Y N consent form completed

FORM A

1. Household listing form

Would you please list all who live in this household, and their age on their last birthday, including yourself? *Start with the Key Informant—the person who knows the most about the household. Note: a household is a group of people who usually sleep and eat together, with a separate entrance and a common kitchen.*

No. (a)	**All household members** (first names only) *X if Household head* *(b)*	**Age at last birthday** (C)	**Sex M/F** (d)	Injury after 2003 Y or N (e)
**1** Key Informant for HH info	☐ HH head?			
**2**	☐ HH head?			
**3**				
**4**				
**5**				
**6**				
**7**				
**8**				
**9**				
**10**				
**11**				
**12**				

Is this correct that there are ____ persons living in this household and -_____ injured person?

2. *Mortality from January 1 2003 until today? Have there been any deaths in the household since 2003?*

Put I if the death was due to injury and N if the death was not due to injury, and list Age and Sex in the same box THESE ARE 3 NUMBERS IN EACH BOX FOR A DEATH

	2003	2004	2005	2006	2007	2008	2009	2010	2011	2012	2013	2014
**1**												
**2**												
**3**												
**4**												
**5**												

FORM B

Injury Definition: An intentional or unintentional physical event that requires medical care and or intervention and results in loss or reduction in normal activities for a while or (or should have received care, which may not have been possible)

.

3. Demographic information on injured persons or injured deceased household members

			skip
3.1	**For which person in part 1 is this information? (number)_____________ *Complete a separate form for each injured persons list in the household listing. If a person has been injured more than once during the time period, fill out a separate form for each of the injuries.***		
Information about the injury and the injured person			
3.2	Age (AT PRESENT) or at time of death	_____________years	
3.3	Marital status (AT PRESENT)	1. Never married<br>2. Currently married<br>3. Separated<br>4. Divorced<br>5. Widowed	
3.4	Education	1. None<br>2. Some primary school;<br>3. Completed primary school<br>4. Completed Secondary high school<br>5. Attended university	
3.5	Current or last job	1 Business<br>2 Government employed<br>3 Self-employed, own business or farming<br>4 Student<br>5 Keeping house/homemaker<br>6 Retired<br>7 Unemployed (health reasons)<br>8 Unemployed (other reasons)<br>9 Other (specify) _______________	
3.6	Year the injury occurred	_____________________ year ***This is a KEY Question!***	
3.7	Location of the injury	1 House<br>2. Farm<br>3 Open public space, street or square<br>4. As a prisoner<br>5 Office, or school<br>6 Industrial site, factory or shop<br>7. In a vehicle on a public road<br>8 Travelling by airplane or train<br>9 Other location (specify)_______________	
3.8	Current status of injured person	1. Alive, and functioning normally<br>2. Alive with reduced function<br>3. Dead,	If1,2 skip to next section➔
3.9	If died….	Time after the injury when the death occurred<br>_______months or ________ years	
3.10	If dead….	1. Death occurred from injury<br>2. Death may have occurred from injury (not sure)<br>3. Death occurred was unrelated to the injury	

4. *Nature of injuries after 1 January 2003 (use separate pages for part 4-7 for each injury)*

This section has 3 parts.<br>Section 1 is asks about what parts of the body were injured and what were the physical results of the injury<br>Section 2 concerns civil injuries, or "unintentional violence"—injuries not from conflict or warfare<br>Section 3 is about injuries that occurred from violence, conflict or warfare, that is "intentional injuries"			
Section 1, Type and consequences of injuries			
4.1	What parts of the body were injured? *More than one possible*	1 Head<br>2. Face<br>3 Neck<br>4. Chest<br>5 Back or spine<br>6. Abdomen<br>7. Upper arm<br>8. Lower arm<br>9. Hand<br>10. Hip<br>11. Pelvis<br>12. Upper leg<br>13. Lower leg<br>14. Foot	
4-2	What was the nature of the injury?	1 Amputation unilateral limb<br>2 Amputation bilateral limbs<br>3 Amputation thumb<br>4 Amputation finger or toe<br>5. Dislocation joint<br>6 Traumatic Brain Injury mild<br>7. Traumatic Brain Injury severer<br>8. Spinal cord lesion<br>9 Burn lower airway (smoke)<br>10. Burn < 20% skin<br>11. Burn >20% skin<br>12. Fracture<br>13. Foreign Body<br>14. Multiple injuries<br>15. Other / Don't Know	
Section 2, Civil injuries, or unintentional injuries			
4.3	Type of injury	1. Electrical injury<br>2. Fall<br>3 Crush injury<br>4 Nerve injury (including spinal cord)<br>5 Muscle, joint or tendon<br>6 Eye injury<br>7 Poisoning<br>8 Mechanical<br>9 Explosion (unintended violence)<br>10 Intentional such as suicidal intent<br>11 Intentional such as domestic violence<br>12 Others _______________<br>13 Transportation related if ***yes*** ➔next question	
4.4	Was this a work-related injury	1 Yes 2 NO	
4.5	Transportation related trauma (if involves a military vehicle use intentional violence section)	1. In private car<br>2. Taxi<br>3. Public bus<br>4. Motor cycle or bicycle<br>5. Pedestrian injury<br>6 Airplane<br>7. Train<br>8. Boat<br>9 Other ___________________	

Section 3. Intentional injuries, from violence conflict or war			
In this section, participants will be asked if the injury was caused by a ***criminal activity*** *or* ***war-related****.* ***Criminal*** *means events caused during robbery, assassinations, kidnapping, hijacking or assaults likely carried out for personal reasons and the individual was specifically targeted or a bystander to a specifically targeted attack.* ***Conflict related*** *means injured by in the course of a wider indiscriminate intentional violence whether by militias or organized military and whether for sectarian or in the course of organized military operations.*			
4.5	Type of injury	1. Gunshot	

		2. Penetrating wounds, (including stab wounds)<br>3. Shell injuries, fragment<br>4. Blast/explosive injury<br>5. Burns<br>6. Torture (prisoners)<br>7. Other trauma<br>(specify)__________________________<br>_________________________________	
4.6	Who do you think was responsible for the injury	1 Criminals,<br>2 Iraq police or army<br>3 Coalition forces,<br>5 Militias or sectarian groups<br>6. Self<br>7. Family members or friends<br>8 Other (specify) _________________<br>9 Unknown,	

5. Treatment of the injury

			skip
Initial treatment of injury			
5.1	After injury, where did the person receive medical assistance?	1 .... Hospital<br>2 .... PHC clinic<br>3 .... Clinic at work<br>4 .... Other<br>5 .... Did not receive care	
Care at hospital. Skip this section if no hospital care			
5.2	Did the patient require surgery or a surgical procedure?	1. During the procedure the patient was ....given a general anesthetic?<br>2.. During the procedure the patient was ....awake (local anesthetic)<br>3. No procedure or surgery was done	
5.3	How long were hospitalizations (weeks)	1 no hospitalization<br>2 first hospitalization_____ weeks<br>2. second hospitalizations____ weeks<br>3. third hospitalization_____ weeks	
Following initial treatment			
5.4	Did the injured person require physiotherapy or rehabilitation post injury?	1 .... Yes<br>2 .... No	
5.5	In total, how much do you estimate your household spent on treating the injury of this household member up until the present time?	Dinars___________________ *or*<br>*US$* ______________________	
5.6	Were you able to meet the financial needs of this hospitalization and any care that was required afterwards?	1 .... Own savings<br>2 .... Help from family<br>3 .... Borrowed money<br>4 .... Sold assets<br>5 .... All care was free<br>6 .... Other	

6. Disability resulting from the injury

	**A disability is defined here as a limitation to the ability to carry out normal activities, and which came about from the injury described in the previous parts of the questionnaire**		***Skip***
Disability			
6.1	Did the injured person suffer any disability that affects the ability to do normal activities for some days?	1....................................... Yes<br>2....................................... No	***If no ➔7.1***
6.2	If ***yes***, how long did this last?	________Days *or*<br>________Weeks *or*<br>________Months *or*<br>________Years *or*<br>☐ Disability is continuing	
**How did this disability affect the person during the time it lasted (0r continues to affect the person? *Consider the areas below***			
6.3	Self-care: Ability to care for self (bathing, feeding).	1.. Yes<br>2.. Partly<br>3.. No	
6.4	Walking	1 .... Difficult to walk around the house<br>2 .... Able to walk around the house, but nothing more<br>3 .... Able to walk outside up to ½ km<br>4 .... Can walk more than ½ km	
6.5	Stair climbing	1 .... Able to climb stairs without difficulty<br>2 .... Can climb stairs unassisted but only with difficulty<br>3 .... Unable to climb stairs without assistance	
6.6	Other type or extent of disability resulting from the injury<br>1. Blindness<br>2. Deafness<br>3. Continuing mild pain<br>4. Continuing severe pain<br>5. Fears, anxiety or emotional instability<br>6. Emotional changes that prevented usual activities or employment<br>7. Other conditions (Specify)____<br>____		

7. Socio-economic consequences from the injury

No.			***Skip***
Disability			
7.1	Did the usual household income decline (money coming in) after the injury? *(for injuries lasting at least a week)*	1................................ Yes<br>2................................ No	
7.2	Did the usual household food consumption decline after the injury? *(for injuries lasting at least a week)*	1................................ Yes<br>2................................ No	
7.3	Did the injured person or the family have to borrow any money to make up for loss of income of the injured person?	1................................ Yes<br>2................................ No	
7.4	***If Yes,*** How much was borrowed?	Dinar_____or US$_____	
7.5	Did any member of the family change his/her usual activities to undertake the activities of the injured person (e.g. farming, housekeeping, work)	1................................ Yes<br>2 No	
7.6	Did the injured person require the assistance of people (relatives or others) from outside to provide assistance in the house or accompanying them to treatment site, etc.	1................................ Yes<br>2 No	
7.7	Did the injured person suffer negative social consequences or stigma as a result of the injury?	1................................ Yes<br>2..	
