# Supplementary material for: Conflict-related intentional injuries in Baghdad, Iraq, 2003–2014: A modeling study and proposed method for calculating burden of injury in conflict
Source: PLoS Med. 2021 Aug 5;18(8):e1003673. doi: 10.1371/journal.pmed.1003673 (PMC8376016; doi:10.1371/journal.pmed.1003673)
Supplement: Attachment — Submitted filename: Response to Plos Med reviewers Jensen paper-adf_AH_Clean.docx [file pmed.1003673.s006.docx]

PLOS Medicine reviews of the Jensen paper

17 July 2020

Response to editors

| **Editors** |  |
| --- | --- |
| 1. We’d suggest revising the title according to PLOS Medicine’s style, ideally this should include some indication of the study design as part of the title after a colon | *We appreciate this suggestion and have revised the title of the manuscript in accordance with the suggested format. We have changed the title to:*  “Calculating the burden of conflict-related injuries and disabilities in Baghdad, 2003-2014: A modeling study and proposed method for calculating burden of injury in conflict” |
| 2. Please structure the abstract using the PLOS Medicine headings (Background, Methods and Findings, Conclusions) | *We have altered the abstract to adhere to the suggested formatting guidelines.* |
| 3. In the last sentence of the Abstract Methods and Findings section, please describe the main limitations of the study’s methodology. | *Per the suggestions we have added a statement of limitations to the abstract under the Methods and Findings section. Our study has several limitations. There is a possibility of recall bias among the survey participants with regard to the reporting and attributing of injuries. Second, we have no data past the time of the interview and this required the assumption that individuals with ongoing disability at the end of data collection would not recover, thus possibly counting more disability for injuries occurring closer to the end of the study. Additionally, deaths could be attributed incorrectly when data was incomplete possibly resulting in an underestimation of the total burden of injury.* |
| 4. Please include a short, non-technical author summary | *We appreciate the opportunity to provide this and make our work more accessible; this has been added to the manuscript for resubmission. We will avoid placing the summary in its entirety here in our responses, however it is included in the resubmitted manuscript.* |
| 5. We would suggest revising the quantitative estimates in the abstract so that these are clearer and relate more precisely to the estimates given in the main text, i.e. a point estimate (of central tendency) with uncertainty intervals/confidence intervals | *We would like to present our findings in the most consistent and accessible way possible. We thank you for the suggestion and have made the findings section of the abstract more consistent with the body of the manuscript.* |
| 6. Ideally, please update the in text citation callout format (this should be sequential numerals in square brackets) – if using referencing software this should be fairly quick and easy. | *We have made the suggested changes in the manuscript* |
| 7. Please clarify in the paper if the analytical approach used here corresponded to one laid out in a prospective protocol or analysis plan. Please state this (either way) in the methods section. If a prospective analysis plan was used in designing the study, please include the relevant prospectively written document with your revised manuscript as a Supporting Information file to be published alongside your study and cite it in the Methods section. A legend for this file should be included at the end of your manuscript. If no such document exists please make sure that the Methods section transparently describes when analysis ere planned and when or why any data-driven changes to the analysis took place. In either case, including those made in response to peer review comments – should be identified as such in the Methods section of the paper with rationale. | *The formal written analysis plan was created after data collection. Please see the attached proposal, which details how we performed the “pull calculations” to work out the methodology, prior to our full analysis with bootstrapping and varying disability weights.* |

Response to reviewers

| **Reviewer #1 (statistical review)** |  |
| --- | --- |
| 8. The general approach is fine 9. I commend the authors for using bootstrapping and for making their code available | *Thank you very much for your comments, we are happy to do whatever is necessary to make these methods reproducible and accessible.* |
| 10. It would be better to calculate life expectancy at time of injury or death. The authors use either a worldwide figure or one specific to Iraqis, but both of those are calculated at birth.  The life expectancy of an adult is longer than the life expectancy at birth minus current age.   If this can't be done here, some mention should be made of why not. | *We understand and appreciate the careful attention to life expectancy given the value’s importance to the calculations in this manuscript. In our primary analysis, we calculated life expectancy from the age of death, rather than life expectancy at birth. Since two reviewers had concerns about our methods here, we agree we need to add some clarity surrounding this question. Where the sentence previously read, “YLLs were calculated by multiplying deaths by the life expectancy at age of death based on a GBD standard life table for estimating premature mortality.” The sentence now reads: “YLLs were calculated by multiplying deaths by the life expectancy at age of death based on a GBD standard life table for estimating premature mortality, rather than an Iraqi-specific life expectancy.” Additionally, where the last sentence in the paragraph read: “Because life expectancy represents the aspirational healthy life span for all individuals, we followed the GBD methodology of selecting the lowest observed death rate for any age group in countries of more than 5 million in population, or 86 years.” We have removed the 86 years figure from the sentence so it now reads: “…we followed the GBD methodology of selecting the lowest observed death rate for any age group in countries of more than 5 million in population.” We hope this will add some clarity to the fact that our primary calculations relied on a life table that used life expectancy at age of death, or injury if indicated, rather than life expectancy at time of birth. The Iraqi life expectancy utilized in the sensitivity analysis is based on a life expectancy at time of birth, rather than a life table. However, to our knowledge no country-specific life table is available and use of this figure likely adds to the reproducibility of the results in different environments as World Bank estimates of this type are easily available for nearly all countries, whereas country specific life tables could be cumbersome to create or obtain.*  *Our analysis plan includes a “Pull Methodology” with worked examples of both of these calculations for subject 778 who died of a gunshot wound at age 24: “*a 24-year-old individual has a maximum life expectancy of 86.956 years. Thus, this individual contributed 62.96 YLLs to the overall burden of injury for Baghdad between 2003 and 2013. …The most recent World Bank calculation of life expectancy for Iraq puts the life expectancy at 69.4 years.^11^ Repeating this calculation utilizing country specific life expectancy results in 45.4 YLL by this individual.” |
| 11. Choosing 86 years for life expectancy seems wrong. I think the Iraqi figure used in the sensitivity analysis should be used in the main analysis. (But see above). | *We understand that this is a contentious point. With regard to the specific use of 86 years, please refer to the previous reviewer comment regarding removal of this specific figure for added clarity. With regard to the use of a standard life table rather than an Iraqi-specific life expectancy, we believe this is as much a values based decision as an epidemiologic one. The reasons for using a standard life table this analysis are twofold: first use of the Iraqi life expectancy would undervalue an Iraqi life as compared to a life from a nation with a longer life expectancy. Second, use of a standard publicly available life table will increase the ease of reproduction of the primary analysis by other authors in other populations.* |
| 12. The two assumptions in the first paragraph both seem dubious. | *We apologize but we are unclear to which assumptions you are referring. If you could be more specific we would be happy to address your concerns.* |
| 13. Were there any differences between the 179 respondents and the 46 who refused? | *The 46 individuals who were excluded from the initial 225 were not individuals who refused to participate in the study. They were individuals whose family members participated in the study but lacked certain data points necessary to perform the planned analysis. No mechanism of injury was overrepresented in this group, except for burn injuries of which there were very few in the sample overall. We elected to address this in what was previously entitled the results section, but understand that inclusion within the methods could make it clearer. In light of this, we have elected to move the paragraph describing these individuals into the methods section of the manuscript.* |

| **Reviewer #2** |  |
| --- | --- |
| 1. It is stunning that outright deaths constituted the vast majority of years lost.  This will be surprising to many and is important for thinking about the costs of conflict and in future eras the costs of reparations. It is rare that a second analysis of a survey contributes something new and important, this is an exception. | *Thank you for acknowledging the importance of our findings.* |
| 2. There is a strong tendency for DALY analyses to come from large (e.g. DHS, MICS) datasets that were collected for some other purpose.  This is a great example for a dataset collected for exactly this purpose (in this case, the burden of intentional injuries). | *Thank you again for this positive assessment.* |
| 3. One big criticism is that the discussion and conclusions read like an advertisement for DALY-type analyses but the results argue the opposite. A simple mortality survey is much easier to do than this complicated interview, asking the researchers to make difficult medical assessments (thus all interviewers were physicians) and bootstrap analyses.  A simple mortality survey is closer to the cause of the problem in terms of the causal pathway.  A simple mortality survey will likely have more political effect, be easier for the layperson to understand, and is closer in time and mechanism to all accountability processes like an ICC trial.   If 90% of burden came directly from death, this study screams that we should focus on getting local professionals to focus on the easier more influential method.  The issue is, perhaps with the Uyghurs, or the Rohingya, or other crises the Baghdad experience is not the norm.   Exploring that in other settings seems to me the important value of this technique at this moment in history. | *Thank you for your acknowledging the importance of our findings. The results with regard to injury and mortality causal pathways are published elsewhere.to. However, our goal with this paper was to develop more sophisticated war-related epidemiology methods for, what we believe, is a somewhat under-developed field. We would be thrilled to see our methods applied to populations such as the Uyghurs or Rohingya and believe that part of the value of this technique is that it will be valid across multiple populations and multiple types of conflicts.* |
| 4. Methods Rare and great that the interviewers were physicians. | *Thank you for this positive assessment of our methods.* |
| 5. "Because life expectancy represents the aspirational healthy life span for all individuals, we followed the GBD methodology of selecting the lowest observed death rate for any age group in countries of more than 5 million in population, or 86 years.22"   While this might be referenced, it is so profound that it beckons a line or two more explaining what the reference in this case was (e.g. based on 140 countries across the globe or based on the 11 countries in the Middle-east over 5 million population or based on Baghdad in 2000 with some projection model?).   Moreover there is a danger that future people will repeat this process in a completely different setting and the readers will not understand that difference will be because of the assumed life expectancy.   Thus, it would be ideal if in the methods there could be a table of the male and female life expectancies at birth, 10, 20, 30, 40, 50, 60 ,70, 80, ...>85=0    or something like that. | *The reviewer’s point is well taken with regard to the huge importance of decisions made around life expectancy when performing these calculations. There are many complex decisions involved in adopting assumptions surrounding life expectancy. These have been laid out in great detail in the GBD methodology papers, and providing the rationale and methods behind how these values were created is unfortunately somewhat beyond the scope of this paper. We do understand, however, that our language in this paragraph could be confusing by suggesting that we developed a novel life table for this work, when in fact the GBD life table was utilized. We therefore have altered the paragraph in question to make this clearer to our readers.* |
| 6. YLLs have an outsized effect on total DALY calculations, therefore conservative practice required YLLs to be calculated only for individuals who definitively died of their injury."   The implications of this are not covered in the discussion and should be. | *This is an important point and we have added a paragraph to the discussion noting the implications of this fact.* |
| 7. RESULTS - You say, "Forty-six individuals with intentional injury could not be used in the calculation of DALYs for the sample due to missing data points."  So then is the denominator you used to assessing the burden of disease 5148 individuals, or some fraction of that population corrected for the incomplete data (e.g. 175/229 X 5148 would be one extreme while using 5148 as the denominator would be the other extreme)?  Just ignoring those 46 with incomplete data would be markedly under-estimating the burden of injury and might be explaining why outright deaths are the lion's share of DALY's lost.  Perhaps you did a sensitivity analysis (e.g. exclude vs. assume they had lost the average # DALY's as those with complete records....), but if so, I somehow missed it. | *Thank you for your concern on this point as it is important to the results. Certainly, looking at the results for the city as a whole is more useful than looking at the results for just a sample so extrapolation is needed. We calculated this by arriving at the mean number of DALYs in the sample after bootstrapping. Therefore the number of individuals in the sample contributing DALYs varied with each repetition. So the mean number of DALYs that was divided by 5148 to achieve the mean for the sample was generated from 1000 repetitions with repetition and therefore the actual numerator varied on each repetition.*  *In summary we agree that this is a potential area for underestimation. In light of this we have added the following to the limitations paragraph in the discussion.*  *“Lastly, we extrapolated to the population of Baghdad by multiplying the mean number of DALYs, YLLs, and YLDs by the population of the city. The mean numbers were achieved after bootstrapping, but this is a source for underestimation as the individuals excluded from the study for lack of sufficient data to perform calculations may have increased the mean per person. However, it is unlikely that inclusion of these individuals would have altered the UI for the study. “* |
| 8. Discussion "Both the number of injuries and total burden of injury suggest the majority of morbidity and mortality was attributable to small arms rather than blasts or explosions."   Might be worth mentioning that the western press (as reflected by IBC and others) suggested explosions accounted for most violent deaths.  This highlights the advantages of random HH surveys like this. | *We appreciate the fact that the reviewer shares our view that press-based sources of data collection (such as the Iraq Body Count) misattribute the primary driver of deaths to explosions and blasts rather than small arms fire. We attempted to highlight this fact in the fourth paragraph of the conclusions section. Still, to emphasize this point, we have amended the last sentence to read, “Even if shelling is included along with blasts and explosions, results suggest a two-fold underestimation of the burden of intentional injury, highlighting the importance of survey technique such as random household surveys.”, rather than “Even if shelling is included along with blasts and explosions, results suggest a two-fold underestimation of the burden of intentional injury.” We hope that this clarifies and emphasizes this important point.* |
| 9. There are several mechanisms by which this is a likely under-estimate (if cause of death was blurry, excluding injuries where the data was not complete....) and this is not adequately addressed. | *We understand that our methods are certainly capable of providing an underestimate of the total impact of conflict related injuries and deaths, however our focus was on designing a methodology that could be widely applied and felt it best to be as conservative as possible in doing so. However, we have added an additional paragraph in the discussion to better highlight some of the areas that could lead to underestimates.*  *Additionally, we have covered this in the limitations portion of the abstract as well.* |

| **Reviewer #3** |  |
| --- | --- |
| 1. -       "YLLs were calculated by subtracting the age at death from the age the subject might have expected to achieve at birth": This is not correct, and is not consistent with the Methods section in the full text. For calculating YLL, instead of life expectancy at birth, life expectancy at the age of death is used. | *Thank you very much for pointing out this error. The associated sentence has been e*dited for clarity, here and in the text, *to read, “YLDs were calculated with disability weights, and YLLs were calculated by subtracting the age at death from the life expectancy at the age of death.”* |
| 2. I think "Finding" section of the abstract should include a little bit more of the important findings of the manuscript. | *We understand the reviewers concern regarding the value of placing additional findings in the abstract. We believe the most important piece of this work is the methodology rather than the actual findings. However, we have added the following paragraph to the abstract. “Under our methodology, the majority of DALYs lost were attributable to YLLs, rather than YLDs: 4.99 million YLLs lost (95% UI 3.87 million – 6.13 million) versus 616,000 YLDs lost (95% UI 399,000 – 894,000). We also conducted a cause-based analysis to find more DALYs were lost to from gunshot wounds (57%) than any other cause.* |
| 3. Methods -       Self-harm is not included in the study, so they cannot be considered equal to intentional injuries. It seems that the study covers include conflict and terrorism, as well as interpersonal violence. I think the whole manuscript should be checked for consistency. | *Thank you for this note. As a way to work out and demonstrate our methodology we hoped to focus on violent injuries directly resulting from conflict, which are intentional. You are correct that we did not capture self-harm and therefore we have changed “intentional injuries” to “conflict-related intentional injuries” throughout the manuscript to hopefully clear this up.* |
| 4. -       To calculate YLD, both external causes and nature of injuries should be available. It is not clear how a population survey is valid to extract frequency of different types of bodily injuries (nature of injury); non-medical professionals might remember injuries such as fracture in large bones, but usually are not capable to differentiate for instance a vascular injury from other soft tissue injuries. Also, there is a high probability of forgetting separate minor injuries or minor injuries in the context of a multiple trauma. Also, it is not clear how a cross-sectional survey can be used as a valid tool to estimate both prevalence and incidence during a relatively long period of time. I think it is necessary to explain a little more around the survey and validity of its data that have been used as the main source for this study. | *We thank the reviewer for raising these important limitations. As we have acknowledged in the text, “recall bias” might lead to underestimation by precisely one of the mechanisms the reviewer proposes: forgetting minor injuries. We have added the following text on the other limitation the reviewer has highlighted, which is the possibility that non-medical professionals might not accurately recall and report certain types of injuries. Under the second paragraph in the “Strengths and Limitations” subsection of the Conclusions Section: An additional aspect of recall bias is the lay reporting of certain injuries (i.e. by non-medical professionals) might lead to certain types of injuries being more remembered than others (e.g. long bone fractures might be more memorable than soft tissue injuries.* |
| 5. -       It is not clear how the number of fatalities has been estimated through such survey. | *The number of fatalities was based on the survey questions “Did any family member die?” (During the study time frame), and “How did that person die?” By querying family members the survey was able to gain an estimate of past fatalities.* |
| 6. The data are related to a specific period of time and cannot be updated (not sure why not published earlier), but is there any specific reason for authors preference on using GBD 2013 methodology (while GBD methods are continuously updated) | *Thank you for the opportunity to discuss the methods of this work which we believe are its real contribution to the field of conflict epidemiology. Although the GBD study is regularly updated what we set out to demonstrate here is the general approach that has been used in all GBD studies from 2010 to present. The GBD 2013 study is referenced in order to be precise regarding the disability weights utilized in our calculation.* |
| 7. Findings It is better to provide some data on total number of deaths and injuries, before providing estimates for YLL, YLD and DALYs. | *We understand the utility of comparing our results to the more concrete results of deaths and injuries. In light of this we have added the following sentence to the paragraph noting the results from the study population prior to extrapolation to the city. “Eighty eight of these individuals died as a result of their injury and 137 had injuries resulting in some degree of disability.”* |
| 8. Discussion -       Is there any reason for the relatively huge difference between incidence-based and prevalence-based YLDs? Considering the fact that duration of disability is considered in calculations? | *Thank you for the opportunity to further discuss this point, to better illustrate the difference we have added the following paragraph to the discussion regarding the decision to use incidence YLDS for the primary analysis. “The large difference in incidence YLDs and Prevalence YLDs is best illustrated by focusing on those individuals with lifelong disability. If we take a 20-year-old man who sustains a lifelong disability in 2011, with a DW of 0.2 then our proposed incidence based approach would allocate the entirety of the YLDS from his injury (i.e. ~67 years x 0.2) to the year 2011. Using the prevalence YLD approach 0.2 would be added to each year (i.e. 2012, 2013…) for the remainder of the persons expected life expectancy at the time of injury.”*  *While useful for the assessment of effects of diseases on a population over time, we felt the incidence-based approach was more useful for estimating the acute effects of conflicts in the short term. There is certainly a role for following populations over time, however we thought that for this study of conflicts there was added value in the incidence-based approach as it allows a better picture of the effects of specific events.* |
| 9. There is no comparison with other studies; the estimated at least could be compared with the most recent GBD estimates for the same cause and same period of time | *Given that we are proposing a new methodology for calculating conflict related burden of injury we understand the value of comparing our results to other accepted measurements. Therefore, we have added the following paragraph to the discussion under the “Total DALYs Lost” heading: The end goal of all conflict epidemiology works is to better understand the burden placed on a population by conflict. We believe these methods can provide a more complete picture of the impact of a conflict on a population than previous methods. For comparison our previous works have described the impact of conflict related intentional injuries in Baghdad in terms of injuries per 1000 years of exposure (5.0 95% CI 4.3 – 5.6). The toll of the conflict has also been expressed for the nation as a whole as being 4.55 deaths per 1000 person years (95% UI 3.74 -5.27) or in terms of excess deaths attributable to war as 405,000 excess deaths for the country of Iraq (95% UI 48,000 – 751, 000). While all of these represent accepted methods for describing the impact of conflict on a population, we developed this methodology in order to provide a picture of both the morbidity and mortality of a conflict with a single method and measurement.* |
