# Supplementary material for: Conflict-related intentional injuries in Baghdad, Iraq, 2003–2014: A modeling study and proposed method for calculating burden of injury in conflict
Source: PLoS Med. 2021 Aug 5;18(8):e1003673. doi: 10.1371/journal.pmed.1003673 (PMC8376016; doi:10.1371/journal.pmed.1003673)
Supplement: Attachment — Submitted filename: Response to Plos Med reviewers Jensen paper_November.docx [file pmed.1003673.s007.docx]

PLOS Medicine reviews of the Jensen paper

15 November 2020

Response to editors

| **Editors** |  |
| --- | --- |
| 1. Please place the Author Summary after the Abstract | *Thank you very, much for this edit we have made this change.* |
| 2. Please use standard PLOS Medicine subheadings: (Why Was This Study Done? What Did the Researchers Do and Find? What Do These Findings Mean?) | *Thank you very much for the advice, we altered the headings to be consistent with the authorship guidelines.* |
| 3. Please use bullet points under the 3 subheadings | *We have made the alteration in the author’s summary to conform to the guidance.* |
| 4. Please include summary participant/household demographics | *We appreciate the suggestion and have added summary household demographics from the source study (Lafta et al.) to the methods section with the following text: “Nine-hundred households were visited and information was collected on 5,148 persons. This encompassed individuals who were living and those who had died from their injuries. The average household size was 5.72. The age breakdown was as follows: 12% (n=607) were under the age of 5, 44.2% (n=2,274) were under 19, and 7.2% (n=368) were over the age of 60. Household composition was evenly divided between males and females.”* |
| 5. On line 87 please define DALYs at first use | *We appreciate the suggestion and have made the requested change.* |
| 6. Please move citations before punctuation and remove spaces within the square brackets. | *We appreciate the suggestion and have made the requested changes to the citation format*. |
| 7. Line 293 mention if consent was informed | *We appreciate the opportunity to clarify this point. Informed consent was obtained verbally from the study participants.* |
| 8. Line 370: Rename section Discussion | *The section previously entitled Conclusions has been renamed Discussion* |
| 9. Line 370: Organize Discussion as short clear summary of article findings, what study adds to existing research and why it differs from previous research, strengths and limitations of the study, implications and next steps for the study, implications and next steps for research, clinical practice, and or policy, one paragraph conclusion | *We appreciate the opportunity to organize our work in order to better adhere to the preferred organization of the discussion section for the journal. We have significantly reorganized this section to better fit the suggested framework.* |
| 10. Include study questionnaires as supporting information files | *The study questionnaire has been named Questionnaire S1 and submitted as a supporting information file.* |
| 11. Rename the analysis plan S1 text and update reference line at 197 | *The Analysis plan has been renamed as S1 Analysis Plan* |

Response to reviewers

| **Reviewer #1** |  |
| --- | --- |
| “The authors have addressed my concerns and I now recommend publication.” | *We appreciate the reviewer’s time in reviewing our work, and his recommendation for publication.* |

| **Reviewer #4** |  |
| --- | --- |
| 1. “Disability…is a persistent and socially significant problem especially among communities who have gone through major conflict. The issue is also neglected and hence the paper is a welcome addition to the literature.” | *We appreciate the reviewers support and agree that the impact of injuries on conflict stricken communities is under-appreciated, and we strive with this paper to address this issue.* |
| 2. The section on methods and findings begins with data collection without even a cursory description of the sampling design, household selection, and construction of the sampling frame – all of which are foundational for what is to come- that is extrapolating the results from the 900 households. Why these 900 households are representative of the entire city population is not explained despite being a critical pillar for the credibility of the final estimations. | *We understand that additional details regarding the sampling methodology might be helpful to the reader. In addition we welcome the opportunity clarify that a full accounting of the sampling methods used for this specific data was previously published. However, evidence of the validity of extrapolating our findings to the city as a whole, is extremely important to the study and we have provided a short summary of the sampling technique. “Two-stage cluster sampling techniques were utilized to select 30 random clusters with a start household for each cluster. These clusters were created based on the sampling data for the 14 administrative units within the city of Baghdad. The clusters were created using a probability proportional to estimated size technique. Beginning with the start household, teams used a systematic sample technique including every other house until 30 households were interviewed.”* |
| 3. The YLL and YLD methods are well known and could have been cited to the GBD documents. | *We appreciate the reviewers view on this and welcome the opportunity to clarify our reasons for presenting our methods in this way. While the GBD methodology is well published, we made the decision to use incidence as opposed to prevalence based DALYs which differs from the GBD methods which utilize prevalence based DALY methods. Our primary goal with this manuscript is a clear explanation of our methods in order to allow for application of our methods to future conflicts. In pursuit of this goal we elected to clearly explain how we applied to the methods to this data set and how we altered the calculations to better fit a specific conflict data set. We did attempt to clarify this in our methods to clarify our proposed modifications to these methods. Under the section entitled “*Use of Incidence vs Prevalence DALYs*” we have added the following sentence. “*This represents the main deviation from the GBD methodology for application to a specific conflict data set” (Line 271-272) |
| 4. The study applies standard YLL and YLD methods to the data and that, per se, is not innovative. What maybe innovative is that this well-developed method is applied to Baghdad which had gone through a war. It may be a better idea to emphasize that aspect more than an innovation on the method. | *We appreciate the reviewer’s advice to place more emphasis on application of these methods to the population of Baghdad. In light of this we have made changes to our discussion section to include a specific paragraph on how this differs from previous research that emphasizes the importance of applying these methods to the city of Baghdad and other populations affected by conflict. (Beginning Line 415)* |
| 5. The results are a bit indecipherable since they don’t make reference (Line 381-384) to anything else for the reader to compare – even YLL and YLDs made by GBD in poor countries without a conflict or indeed cities with a lot of urban violence such as Medellin or Lagos. What does 5.6 million DALYs mean in relation to other settings? | *Given that we are proposing a modification to the GBD methodology for calculating conflict related burden of injury we understand the value of comparing our results to other accepted measurements. As to our knowledge this is the first application of this altered methodology to a conflict population it is difficult to make comparisons to other populations.*  *Our hope is to clearly delineate our methods so that future conflicts can be evaluated using the same methodology, thereby giving the conflict epidemiology community the opportunity compare the burden of different conflicts on different populations through the same lens.* |
