# Supplementary material for: Conflict-related intentional injuries in Baghdad, Iraq, 2003–2014: A modeling study and proposed method for calculating burden of injury in conflict
Source: PLoS Med. 2021 Aug 5;18(8):e1003673. doi: 10.1371/journal.pmed.1003673 (PMC8376016; doi:10.1371/journal.pmed.1003673)
Supplement: Attachment — Submitted filename: Response to Reviewers_Final.docx [file pmed.1003673.s008.docx]

PLOS Medicine Response to Reviewers for the Jensen et al. paper

May 2021

Response to Editor

| **Editors** |  |
| --- | --- |
| 1. Please address comments that context for results would be helpful. | *Thank you for your comments, please see number 28 on this document for our comments regarding how we have attempted to show comparison to other methods of calculation.* |
| 2. Please be consistent with the use of hyphenation in conflict-related throughout the text. | *The phrase “conflict-related” has been hyphenated throughout the text for consistency.* |
| 3. Please correct the ethical approval section of the submission form to reflect the methods section of the text. | *We have made this correction to the ethical approval section of the submission form as requested.* |
| 4. Please revise the title according to PLOS medicine style. | *Thank you for this suggestion. We have changed the title of the submission to “Conflict-related intentional injuries and disabilities in Baghdad, Iraq, 2003-2014: A modeling study and proposed method for calculating burden of injury in conflict.”* |
| 5. In the Abstract please expand on the issues with using mortality rate as a primary measure of the population health effects of war. | *Thank you for this suggestion, we have added the following language to the Abstract. “However, mortality only demonstrates part of the burden placed on a population by a conflict, injuries and their resulting disability also have long term effects on a population and are not accounted for in estimates that focus solely on mortality.”* |
| Abstract Methods and Findings |  |
| 6. Please add that this is a modeling study. | *Thank you for this opportunity to offer clarification. We have changed the first sentence of this section to read: “We aim to describe this modeling study in order to offer an innovative methodology for…”* |
| 7. Please provide brief demographic details on the population studied (gender, age, etc.). | *We have added the following 2 sentences to the methods section of the abstract: “The majority of the population (55.8%) were between the ages of 19 and 60. Household composition was evenly divided between males and females.”* |
| 8. Please provide more detail on the data collected during the interviews (e.g., self-reported injury, ability to work) that has informed your findings. | *We have attempted to address the information requested here and in addition have submitted the original survey in its entirety as a supplement. In order to clarify the content from the survey used in our calculations we have added the following sentence to the methods section of the abstract. “The survey questions were answered by the head of household or senior adult present. The questions included year the injury occurred, the mechanism of injury, the body parts injured, whether the injury resulted in any disability and if so, the length of disability.”* |
| 9. Please move the paragraph beginning “Our data come from interviews…” to the beginning of the Abstract Methods and Findings Line 45. | *We have made the proposed change to the order of this section.* |
| 10. Please consider rephrasing to “YLLs were calculated by subtracting the age at death from the expected age of death”. | *We have rewritten this sentence to read, “YLLs were calculated by comparing the age at death to the Global Burden of Disease Study standard life table to calculate remaining life expectancy. Calculations were also performed using Iraq specific life expectancy for comparison”* |
| 11. Please clarify what the expected age of death is based on. | *See above, but we have attempted to clarify that our primary outcome measures were calculated using the GBD global life expectancy table, as well as performed using the life expectancy for Iraq for comparison.* |
| 12. Line 54 - please define UI at first use. Please revise 4.9 million to 4.99 million (as shown on line 59) if this is correct. | *Thank you for identifying this discrepancy. We have corrected it as requested.* |
| 13. Line 59 - there is no need to repeat results; please omit “4.99 million YLLs lost (95% UI 3.87 million – 6.13 million) versus 616,000 YLDs lost (95% UI 60 399,000 – 894,000).” | *We appreciate the advice on presenting our results in a concise manner. We have deleted the initial description of our results as we feel the second instance provides additional valuable information to the reader.* |
| 14. Please begin your Abstract Conclusions with “"In this study, we calculated ..." or similar, to summarize your findings for the reader. | *We are happy to adhere to the journal’s convention and have altered the initial sentence of our conclusion to read, “In this study we propose a methodology for performing burden of disease calculations for conflict-related injuries (expressed in DALYs) in Baghdad from 2003-2014.”* |
| Author Summary |  |
| 15. Within ‘What Do These Findings Mean?’ Please provide a non-technical summary of your results. | *We have opened this section with the following paragraph: “We calculated a burden of injury of 5.9 million DALYs lost due to conflict-related injuries in Baghdad from 2003-2014. These calculations encompass both mortality as well as the effects of injury and disability on the population of Baghdad. We performed these calculations using a variation of the GBD methodology to better reflect the dynamic nature of conflict.”* |
| 16. Please include a final sentence outlining the limitations of your study. | *The authors welcome to opportunity to be clear about the limitations present and have added the following bullet point: “Limitations for this study include the possibility for recall bias amongst the survey participants as well as the possible undercounting of deaths due to injury due to classifying any injuries that did not definitively result in death as YLDs rather than YLLs. Lastly, we did not adjust for changes in the population of Baghdad over the course of the study.”* |
| 17. Please consider re-ordering the first two bullets points. | *What were previously the first 2 bullet points were reordered. These now form the 2^nd^ and 3^rd^ bullet points of this section.* |
| 18. Please rename ‘Background’ as ‘Introduction’. | *We have renamed the section titled ‘Background’ as introduction* |
| 19. Line 115 - please omit sentence beginning ‘This paper reports a new approach…’. | *This sentence has been omitted as requested* |
| Methods |  |
| 20. Please separate your ‘Methods and Findings’ section into separate ‘Methods’ and ‘Results’ sections. | *We welcome the opportunity to better adhere to the structure requested by the journal and have separated these sections and moved the demographics paragraph into Results section.* |
| 21. Please ensure that the study is reported according to the STROBE guideline and include the completed STROBE checklist as Supporting Information. Please add the following statement, or similar, to the Methods: "This study is reported as per the Strengthening the Reporting of Observational Studies in Epidemiology (STROBE) guideline (S1 Checklist).” | *The authors are happy to ensure that our work is presented in the most complete way possible. We have filled out and submitted as a supplementary file the STROBE-cross sectional studies checklist. Additionally, we have added the requested language to the Methods section under the subheading Analysis.* |
| 22. Please ensure that where methods are described elsewhere e.g., references 11 and 19, that the papers cited are open access. If not, please ensure to describe the methodology more thoroughly. | *The authors appreciate the journal’s commitment to ensuring the process of replicating our results is as straightforward as possible. The two papers specifically mentioned in the review (references 11 and 19) are open access articles. All other papers cited in our methods are open access, with the exception of 21 and 22 which are available free of charge through the Lancet.* |
| 23. Please confirm whether the analysis plan was prospective. | *The analysis plan was prospective* |
| 24. Line 237 - please omit ‘per Salomon’ (citation will suffice). | *This change has been made, the line ‘per Salomon’ has been deleted from the text* |
| 25. Line 314 - please quote the actual number/percentage of individuals excluded due to missing data in place of unspecific terms such as ‘nearly’. | *We have changed this sentence to the exact percentage of individuals excluded from the analysis due to missing information.* |
| Discussion |  |
| 26. Please remove subsection headings within the Discussion (e.g., Total DALYs Lost, and so on). | *We are happy to comply with the stylistic preferences of the journal and have removed the subsection headings as requested.* |
| 27. Please begin your Discussion with a short, clear summary of the article's findings. | *We have begun our discussion with the following sentence, “We estimate 5.6 million human disability-adjusted life-years were lost among residents of the city of Baghdad as a result of conflict- related intentional injuries over the course of the U.S. – led coalition invasion of Iraq and subsequent insurgency.”* |
| 28. Line 390 - please take note of the comments from the Academic Editor “to put these numbers (particularly injuries) in some context with previous work--is a lot or not so many? [Although] methods are different from past literature… it would still be helpful to cite findings from conflict situations that are broadly similar for context”. | *We have inserted the following paragraph in our discussion to provide context for our numbers. “Because of the, to our knowledge, novelty of this method of calculation and its application to a conflict specific data set, it is difficult to compare our results with other settings. We did find a study from South Africa where a team used local data combined with GBD techniques to provide an estimate of DALYs lost attributable to interpersonal violence. Matzopoulos et al calculated an estimated 2,605 DALY per 100,000 population were lost to homicide and other interpersonal violence in South Africa [28]. By our estimates, Baghdad lost 5,320 DALYs per 100,000 population in 2008 and 1,390 DALYs per 100,000 population during 2012, the highest and lowest burden of injury years in our data set. These comparisons should be made cautiously however, as the South Africa data relied on modeling to fill in some data gaps and used a hybrid DALY approach, whereas we used an incidence-based DALY approach.”* |
| 29. Stylistically speaking, please add ‘to the best of our knowledge’ or similar to avoid assertions of primacy (e.g., "Because of the novelty of this research", “ours may be the first attempt…”). | *The authors understand the importance of avoiding assertions of primacy and have altered the text to add qualifying language when discussing the role or our work in relation to the global body of work in this subject area.* |
| 30. Please ensure that references are appropriately formatted, and journal titles are appropriately capitalized. | *The references have been reviewed and every effort made to ensure that they follow the journal conventions.* |
| Figures |  |
| 31. Figure 1 please correct DALYs on the Y axis. | *We apologize for this error and have fixed the figure in question, and re uploaded the file.* |
| Supplementary files |  |
| 32. Line 302 - “We have provided the code and data set in our online appendix. “Please ensure that either the relevant file is appended to your resubmission, or that the freely / publicly available location of the data is shared in your manuscript. | *We apologize for the oversight and have uploaded both the data dictionary with the relevant statistical code for our bootstrapping function as well as a csv file containing the data needed to replicate our calculations.* |
